# Decoding the evidence emerging from the studies comparing conduction system pacing with conventional cardiac resynchronization therapy

**DOI:** 10.1093/europace/euag186

**Published:** 2026-08-13

**Authors:** Jacopo Francesco Imberti, Davide Antonio Mei, Giuseppe Boriani

**Affiliations:** Cardiology Division, Department of Biomedical, Metabolic and Neural Sciences, University of Modena and Reggio Emilia, Via del Pozzo 71, Policlinico di Modena, Modena 41121, Italy; Cardiology Division, Department of Biomedical, Metabolic and Neural Sciences, University of Modena and Reggio Emilia, Via del Pozzo 71, Policlinico di Modena, Modena 41121, Italy; Clinical and Experimental Medicine PhD Program, University of Modena and Reggio Emilia, Modena, Italy; Cardiology Division, Department of Biomedical, Metabolic and Neural Sciences, University of Modena and Reggio Emilia, Via del Pozzo 71, Policlinico di Modena, Modena 41121, Italy

**This editorial refers to ‘Conduction system pacing versus biventricular pacing in heart failure with reduced ejection fraction: a meta-analysis of randomized controlled trials’ by P. Karakasis *et al*., https://doi.org/10.1093/europace/euag170.**

**This editorial refers to ‘Conduction system pacing versus biventricular pacing for cardiac resynchronization therapy in heart failure: a systematic review and meta-analysis considering randomized controlled trials and observational studies’ by K. Chauhan *et al*., https://doi.org/10.1093/europace/euag169.**

Cardiac resynchronization therapy (CRT) delivered by biventricular pacing (BiVP) is at present the standard treatment for selected patients with heart failure with reduced ejection fraction (HFrEF) and electrical dyssynchrony, supported by more than two decades of randomized evidence demonstrating reductions in heart failure hospitalization (HFH) and mortality.^1,2^ Conduction system pacing (CSP), including His bundle pacing (HBP) and left bundle branch area pacing (LBBAP), has emerged as an alternative approach for CRT that directly recruits the His–Purkinje system, with the potential to achieve a more physiological pattern of ventricular activation and, in selected patients with bundle branch block, restore physiological conduction by pacing distal to the site of block.^1,2^ It also has the potential to avoid the detrimental effects associated with right ventricular pacing.^3^ However, whether CSP translates into improved clinical outcomes compared with BiVP is still matter of debate.^4^

In this issue of *EP-Europace*, two independent meta-analyses evaluated the currently available evidence comparing CSP with conventional BiVP for CRT, offering complementary perspectives on a similar pool of contemporary data.

Karakasis *et al*.^5^ pooled data from 13 randomized controlled trials (RCTs) including 1320 patients and reported that CSP was associated with a lower risk of the composite endpoint of HFH or all-cause mortality compared with BiVP [odds ratio (OR): 0.51; 95% confidence interval (CI) 0.28–0.93]. However, when the individual components were analysed separately, neither HFH (OR: 0.68; 95% CI 0.36–1.27) nor all-cause mortality (OR: 0.75; 95% CI 0.36–1.58) differed significantly between treatment strategies. Of note, CSP was associated with greater improvements in functional capacity and QRS narrowing, without differences in procedural safety.

Chauhan *et al*.^6^ adopted a complementary approach by including 11 RCTs (1201 patients) together with 14 observational studies, for a total of 4577 patients. In the prespecified RCT-only analysis, no significant differences were observed for all-cause mortality [risk ratio (RR): 0.96; 95% CI 0.45–2.04], HFH (RR: 0.67; 95% CI 0.40–1.11), or procedural complications (RR: 0.83; 95% CI 0.51–1.34), although CSP achieved greater QRS shortening and modest improvements in functional status. When observational studies were included in the sensitivity analysis, the estimated treatment effect became larger, with lower all-cause mortality (RR: 0.74; 95% CI 0.61–0.91) and HFH (RR: 0.64; 95% CI 0.53–0.77) observed in the CSP group.

Although the conclusions of these two meta-analyses may appear different, their findings are in part consistent and primarily reflect differences in study design and analytical approach.

One important difference is the choice of the primary endpoint used to summarize the available evidence. Karakasis *et al*.^5^ considered the composite of all-cause mortality and HFH as the primary outcome, whereas Chauhan *et al*.^6^ focused on the individual disaggregated endpoints. Composite endpoints are frequently used in clinical trials because they increase statistical efficiency when individual event rates are relatively low. However, their interpretation depends on the relative contribution of each component, which is determined by its clinical importance, frequency of occurrence, and whether the treatment exerts a similar effect across all components.^7^ Karakasis *et al*.^5^ reported a significant reduction on the composite endpoint, whereas mortality and HFH were neutral when analysed separately. Rather than indicating conflicting findings, this pattern most likely reflects limited statistical power for the individual components. Given the relatively small number of deaths across the available trials, the positive composite outcome is likely driven predominantly by differences in HFH.

The two meta-analyses also differ in the types of studies included. Karakasis *et al*.^5^ restricted their analysis to RCTs only, whereas Chauhan *et al*.^6^ performed a sensitivity analysis including also observational studies. The inclusion of observational data was associated with larger treatment effects, with both mortality and HFH reaching statistical significance. This observation should not be surprising. Patients selected for CSP in routine clinical practice may differ systematically from those undergoing BiVP (less sick, more likely to survive), introducing residual confounding by indication despite statistical adjustment. Consequently, observational studies may overestimate treatment effects relative to randomized comparisons. Nevertheless, observational evidence should not be dismissed. Such studies are important for evaluating feasibility, implementation, safety, and real-world effectiveness of rapidly evolving technologies such as CSP.^8^ Rather than replacing randomized evidence, they provide complementary information regarding how these techniques perform outside highly selected trial populations.

As summarized in *Table 1*, meta-analyses restricted to RCTs and those incorporating observational studies have distinct methodological characteristics, each with specific strengths and limitations that should be carefully considered when interpreting their findings. For many contemporary cardiovascular interventions, including CSP, well-conducted observational studies should be regarded as a complementary rather than competing source of evidence and interpreted accordingly. However, it is noteworthy that a recent analysis of the medical literature, including 220 systematic reviews and 217 meta-analyses, found that the inclusion of non-randomized studies changed the effect estimates in 35.9% of meta-analyses, with 88.5% of these gaining statistical significance after their inclusion.^9^

**Table 1:** Comparison of meta-analyses restricted to randomized controlled trials vs. those including both randomized and observational studies

	Meta-analysis of RCTs only	Meta-analysis of RCTs + observational studies
Primary objective	Estimate treatment efficacy under controlled conditions	Estimate treatment effects integrating efficacy and effectiveness in real-world practice
Internal validity	Generally highest because randomization minimizes measured and unmeasured confounding	Lower overall, because observational studies are susceptible to confounding, selection bias, and residual bias despite statistical adjustment
External validity	Often limited by restrictive eligibility criteria	Usually greater, reflecting routine clinical practice and broader populations
Risk of bias	Mainly related to randomization process, allocation concealment, blinding, attrition, and outcome assessment	Additional risks include confounding by indication, immortal time bias, selection bias, information bias, time-varying confounding, and residual confounding
Causal inference	Strongest design for establishing causal treatment effects	More limited, since associations may reflect both treatment effects and systematic differences between treated and untreated patients
Clinical heterogeneity	Usually lower	Typically higher, reflecting diverse populations and study designs
Statistical considerations	Relatively homogeneous estimates	Greater heterogeneity: careful bias assessment and sensitivity analyses required
Principal strengths	Highest confidence in causal inference	Greater generalizability, larger sample size, and improved assessment of safety and effectiveness
Principal limitations	Limited generalizability and ability to detect rare events	Residual confounding may influence pooled estimates despite statistical adjustments
Evidence certainty	Usually high (may be downgraded for methodological concerns)	Usually low (may be upgraded in specific conditions, such as large effects and dose–response relationship)

RCT, randomized controlled trial.

The different analytical approaches used in these two meta-analysis further extend to the measures of association used to quantify treatment effects. Karakasis *et al*.^5^ reported odds ratios, whereas Chauhan *et al*.^6^ used risk ratios. Although these measures are not numerically identical, they converge when event rates are low, as in the available CSP trials.

The meta-analysis by Karakasis *et al*.^5^ also provides an informative subgroup analysis according to CSP modality. Despite important procedural differences between HBP and LBBAP, treatment effects remained directionally consistent across both techniques without evidence of significant interaction. Although these analyses are underpowered to determine whether one physiological pacing strategy is superior to another, they support the broader concept that recruitment of the conduction system, rather than the specific pacing modality itself, may represent the principal determinant of clinical benefit.

Both meta-analyses^5,6^ showed consistency regarding secondary outcomes. Across studies, CSP achieved greater electrical resynchronization, reflected by more pronounced QRS narrowing, together with modest but reproducible improvements in functional status, including the New York Heart Association functional class and exercise capacity. The improvement in surrogate measures may support the hypothesis that a more physiological ventricular activation may ultimately translate into better long-term outcomes.

These findings should also be interpreted in the context of how CRT is delivered. The comparison between BiVP and CSP is not simply between two pacing modalities but also between the quality of therapy achieved with each approach. Conventional BiVP may be limited by unfavourable coronary sinus anatomy, suboptimal left ventricular lead position, phrenic nerve stimulation, underlying scar, or incomplete resynchronization.^1,10^ Likewise, the clinical benefit of CSP depends on successful and sustained recruitment of the conduction system. Deep septal pacing without true conduction system capture, lead microdislodgement, or loss of capture during follow-up may substantially reduce the physiological advantages achieved at implantation.^11,12^ These factors likely contributed to the heterogeneous results observed across recent randomized trials, which differed considerably in operator experience, implantation success, and validation of conduction system capture. Equally important, little is known about the long-term stability of conduction system capture. Although acute procedural success is generally high, follow-up data confirming sustained physiological pacing remain limited. Whether a proportion of patients gradually lose effective conduction system recruitment remains largely unknown and deserves systematic evaluation in future studies.

Finally, although the number of RCTs comparing CSP with BiVP has increased substantially over recent years, most studies remain relatively small, reported event rates are low, and follow-up duration varies considerably across trials. Differences in patient selection, CSP modality, operator experience, endpoint definitions, and follow-up duration further contribute to clinical heterogeneity and should be considered when interpreting the available evidence. Implementation of CSP as a standard practice is still incomplete across Europe^13^ and appropriate training of the operators, also with the help of simulators, is a key element for successful CSP procedures.^14,15^

Taken together, the two meta-analyses^5,6^ published in this issue of *EP Europace* provide a consistent message. Although a potential benefit of CSP over BiVP on HFH and mortality cannot be excluded, current evidence remains insufficient to establish clinical superiority. Adequately powered randomized trials with standardized implantation techniques, rigorous confirmation of conduction system capture, and sufficiently long follow-up are needed to determine the effect of CSP on hard clinical outcomes.
